# Compound microbial intelligent membrane fermentation reshapes the virome and reduces various resistance genes in duck litter

**DOI:** 10.3389/frmbi.2026.1897965

**Published:** 2026-09-07

**Authors:** Tiecheng Sun, Guiheng Mei, Junru Mo, Meiqin Li, Yanmei Ma, Mengshi Zhao, Song Peng, Fengqiang Lin, Jilong Chen, Yaxiong Ma, Zhaolong Li

**Affiliations:** 1 Key Laboratory of Animal Pathogen Infection and Immunology of Fujian Province, College of Animal Sciences, Fujian Agriculture and Forestry University, Fuzhou, China; 2 Gut Microbiome Research Center, Institute of Animal Husbandry and Veterinary Medicine, Fujian Academy of Agricultural Sciences, Fuzhou, Fujian, China

**Keywords:** antibiotic resistance genes, biocide resistance genes, compound microbial intelligent membrane fermentation, duck litter, metal resistance genes, metavirome, virulence factors

## Abstract

Contamination of duck litter is a major contributor to frequent disease outbreaks in the scaled dry-litter rearing system, posing serious threats to duck health. Although compound microbial intelligent membrane fermentation has multiple advantages, its impacts on the litter virome and resistome remain poorly characterized. This study employed metaviromics to examine the effects of compound microbial intelligent membrane high-temperature fermentation on duck litter from four treatment groups: deep-layer fermentation (DL-D), shallow-layer fermentation (DL-S-NAF), shallow-layer without fermentation (DL-S), and shallow-layer from an antibiotic-using farm without fermentation (DL-S-ANF). We observed that fermentation treatments (DL-D and DL-S-NAF) significantly reduced RNA viral abundance, with a corresponding decreasing trend for DNA viruses. Fermentation treatments also markedly decreased the loads of antibiotic resistance genes (ARGs) including *sul1*, *sul2*, *tetB(P)*, and *qacEdelta1*; metal resistance genes (*MRGs*) including *arsC*, *arsM*, *merA*, *copR*, and *corR*; biocide resistance genes including *mdeA*, *actP*, *smdB*, *cpxA*, and *galE*; and bacterial virulence factors including g*groEL2* and *lirB*. In duck litter, viral communities were dominated by *Uroviricota* and *Nucleocytoviricota* (DNA viruses) and *Pisuviricota* and *Lenarviricota* (RNA viruses), with *Caudoviricetes* and *Megaviricetes* as the predominant DNA virus classes. Single-sample metavirome analysis identified 34 viral operational taxonomic units (vOTUs) and 343 viral genes. *ARG* abundance followed a hierarchical trend of DL-D < DL-S-NAF < DL-S < DL-S-ANF, while MRG abundance showed DL-D < DL-S-NAF < DL-S-ANF < DL-S. Compound microbial intelligent membrane fermentation effectively reduced both DNA and RNA viral abundance, diminished resistance gene loads, and attenuated virulence factors in duck litter. These data indicate that RNA viruses, particularly *Pisuviricota*, are sensitive bioindicators for ecological health assessment. Additionally, this antibiotic-free fermentation system may provide an important strategy for curbing dissemination of antimicrobial resistance at its source.

## Introduction

### Importance of duck production and dry-litter system

Ducks occupy a significant position in China’s waterfowl farming industry due to their rapid growth, high lean meat percentage, and adaptability to roughage feeding (Lyu et al., 2023). The dry-litter rearing model, also referred to as thick-litter floor rearing, involves housing ducks on a built-up litter layer (typically 10–15 cm) composed of materials such as sawdust, rice husks, or crushed straw throughout the entire growth cycle. This model is currently the primary method for large-scale meat duck production (Lin et al., 2023). The dry-litter system facilitates centralized manure treatment, as waste is concentrated within the house litter, thereby preventing direct pollution of external water bodies (Tao et al., 2014). The mixture of manure and bedding can be recycled as an organic fertilizer resource, aligning with the environmental requirements of modern intensive farming (Zhao et al., 2015).

### Environmental and disease problems of litter accumulation

As waterfowl, ducks have high water consumption during growth, and their excreta possess high moisture content, which readily leads to damp and compacted litter (Liu et al., 2024). Moist litter creates an ideal breeding ground for pathogenic microorganisms including viruses, *Escherichia coli*, and *Salmonella*, and parasites (Dozier et al., 2005). Furthermore, the decomposition of manure within the litter generates substantial amounts of harmful gases such as ammonia (NH_3_) and hydrogen sulfide (H_2_S). Inadequate ventilation allows these gases to irritate the respiratory mucosa of ducks, compromising its natural barrier function, which can cause respiratory diseases and reduce immune competence (Yu et al., 2016; Naseem and King, 2018).

### Current limitations of litter management practices

Direct contact between duck flocks and manure or litter establishes a fecal–oral transmission route, significantly increasing the risk of infections with pathogens, leading to various diseases including digestive diseases (enteritis and viral hepatitis) and parasitic diseases (Lyu et al., 2023; Li et al., 2024). The standardized, large-scale production of meat ducks necessitates thorough removal and replacement of litter, followed by stringent washing, disinfection, and resting of the housing after each flock. However, owing to the limited availability and rising costs of litter materials, many farmers only perform simple sun-drying and tilling of the existing litter after each batch (Sargeant et al., 2019), which results in frequent disease outbreaks, increased antibiotic use, and decreased farming efficiency in duck enterprises, particularly in southern China (Liu et al., 2024). Therefore, development of efficient manure treatment technology that decreases fecal pollution, and improving the in-house environment to reduce disease risks has become an urgent requirement for the healthy and sustainable development of the meat duck industry (Bernal et al., 2009; Fang et al., 2024).

### Introduction of microbial membrane fermentation technology

The compound microbial intelligent membrane high-temperature fermentation technology is currently recognized as one of the primary methods for treating litter and converting manure into valuable resources. This technology integrates three core components: compound microbial agents, functional membranes (nano-membranes and molecular membranes) to cover the compost pile, and an intelligent control system that regulates the high-temperature fermentation process, thereby effectively transforming livestock manure and bedding into stable organic fertilizers or recycled litter resources (Larney and Blackshaw, 2003; Johansen et al., 2013). Here, the term fermentation is used in the broader biotechnological sense, encompassing both aerobic and anaerobic microbial bioconversion. In terms of its mechanism of action, the functional molecular membrane within this system facilitates the evaporation of water vapor while effectively containing malodorous gases, including ammonia and hydrogen sulfide, preventing their release into the surrounding environment and comprehensively addressing odor issues on farms. Additionally, the entire manure treatment process is conducted within an enclosed membrane, which prevents the leachate contamination of soil and groundwater commonly associated with traditional composting due to rainwater runoff, resulting in notable environmental benefits. The bioheat generated by microbial fermentation consistently maintains the pile temperature within a range of 80-85°C, which effectively inactivates various pathogens and weed seeds present in the duck litter, achieving a high level of harmless treatment (Wang et al., 2011; Qian et al., 2014; Chan et al., 2016). Implementing this system for treating litter from dry-litter duck rearing and reintroducing the processed litter back into the duck houses can block disease transmission pathways at the source and significantly enhance the overall health of the duck flock. Furthermore, the entire fermentation process requires no turning or construction of dedicated factory buildings, allowing one operator to manage multiple fermentation piles simultaneously, thereby substantially reducing labor intensity and manpower costs in the farming process (Maeda et al., 2011; Caceres et al., 2018).

### Knowledge gap and study objective

Recent advances in metaviromics have enabled unprecedented resolution of viral community structure and function in complex environmental samples. Previous studies have demonstrated that animal farming environments serve as significant reservoirs of antibiotic resistance genes (*ARGs*) (Zhou et al., 2019) and that litter treatments profoundly influence microbial community composition and antimicrobial resistance profiles. However, comprehensive multi-dimensional assessments integrating viral community composition, *ARGs*, metal resistance genes (*MRGs*), biocide resistance genes (*BRGs*), and virulence factors (*VFs*) in duck litter remain lacking. Furthermore, the efficacy of intelligent membrane fermentation technology in mitigating these biological risks has not been systematically evaluated using metaviromic approaches (Zhou et al., 2019).

In this study, we aimed to address these knowledge gaps by first profiling the DNA and RNA metaviromes of duck litter across four distinct treatment groups using high-throughput metaviromic sequencing, and then analyzing the taxonomic composition and viral community structural changes following compound microbial intelligent membrane fermentation. We further sought to determine the abundance changes of ARGs, MRGs, BRGs, and VFs across the different treatment groups, and to perform single-sample in-depth functional annotation of the viral metagenome for high-resolution characterization of the resistome and virulome. Together, these efforts were aimed at providing foundational data for understanding viral diversity in duck litter and optimizing litter treatment in poultry production.

## Materials and methods

### Sample collection

Duck litter samples were collected from four separate commercial duck farms (n = 5, total N = 20 samples). Farm 1 employed deep-layer fermentation without prophylactic antibiotics (DL-D). Farm 2 employed shallow-layer fermentation without prophylactic antibiotics (DL-S-NAF). Farm 3 was a conventional farm without fermentation or prophylactic antibiotics (DL-S). Farm 4 was a conventional farm using prophylactic antibiotics without fermentation (DL-S-ANF). All samples underwent high-throughput metavirome sequencing for both DNA and RNA viral communities.

### Sample preprocessing and viral enrichment

Duck litter samples (50 g per replicate) were homogenized and suspended in 200 mL of SM buffer (50 mM Tris-HCl, 100 mM NaCl, 8 mM MgSO_4_, pH 7.5). The suspension was shaken at 200 rpm for 30 min at 4°C and then centrifuged at 5,000 × g for 10 min at 4°C to remove large particulate debris. The supernatant was sequentially filtered through 0.45 μm and 0.22 μm polyethersulfone (PES) membrane filters (Millipore, Burlington, MA, USA) to remove bacterial cells and enrich for viral particles. For RNA virus samples, the filtrate was treated with DNase I (20 U/mL; TaKaRa, Kusatsu, Japan) at 37°C for 30 min to remove free DNA, followed by heat inactivation at 65°C for 10 min. The viral particles in the filtrate were then concentrated by polyethylene glycol (PEG) 8000 precipitation (10% w/v PEG 8000, 1 M NaCl) at 4°C overnight, followed by centrifugation at 12,000 × g for 30 min. The resulting viral pellet was resuspended in 500 μL of SM buffer for subsequent nucleic acid extraction.

### Fermentation conditions

For DL-D and DL-S-NAF groups, the compound microbial agent (composed of *Bacillus* subtilis [2 × 10^9^ CFU/g], *Bacillus licheniformis* [1 × 10^9^ CFU/g], *Lactobacillus plantarum* [5 × 10^8^ CFU/g], and *Saccharomyces cerevisiae* [5 × 10^8^ CFU/g], which were obtained from Gut Microbiome Research Center (Institute of Animal Husbandry and Veterinary Medicine, Fujian Academy of Agricultural Sciences), and was applied at a rate of 1 kg per ton of litter material. The deep-layer fermentation piles (DL-D) were constructed to a height of 2.0–2.5 m with a base width of 3.0–4.0 m, while shallow-layer piles (DL-S-NAF) were 0.8–1.2 m in height. Piles were covered with the functional intelligent membrane and fermented for 15–18 days. The intelligent control system maintained aeration at 0.3–0.5 m³/min per ton of material, with temperature monitored daily at three depths (surface, middle, and bottom). Initial moisture content was adjusted to 55–65%.

### DNA and RNA extraction

Total DNA was extracted from the duck litter samples using the E.Z.N.A. Viral DNA Kit (Omega Bio-tek, Norcross, GA, USA) according to the manufacturer’s instructions. DNA samples meeting quality standards (OD_260_/_280_ = 1.8–2.2, OD_260_/_230_ ≥ 2.0) were selected for sequencing library construction. Total RNA was extracted using TRIzol reagent (Invitrogen, Carlsbad, CA, USA) following the manufacturer’s protocol, and genomic DNA was removed using DNase I (TaKaRa, Kusatsu, Japan). RNA quality was assessed using a 2100 Bioanalyzer (Agilent Technologies, Santa Clara, CA, USA), and quantification was performed using an ND-2000 spectrophotometer (NanoDrop Technologies, Wilmington, DE, USA). RNA samples meeting quality standards (OD_260_/_280_ = 1.8–2.2, OD_260_/_230_ ≥ 2.0) were selected for sequencing library construction.

### DNA and RNA library construction and high-throughput sequencing

DNA metagenomic sequencing libraries were prepared using the Illumina TruSeq Nano DNA Sample Preparation Kit (Illumina, San Diego, CA, USA), with 1 μg of total DNA as input. The procedure comprised DNA end-repair, A-tailing, and adapter ligation with NGS-indexed tags, adhering to standard next-generation sequencing (NGS) protocols. Size selection targeted approximately 400 bp fragments using a 2% low-melting-point agarose gel, followed by 15 cycles of PCR amplification with Phusion DNA Polymerase (New England Biolabs, Ipswich, MA, USA).

RNA metatranscriptomic sequencing libraries were prepared using the TruSeq RNA Sample Preparation Kit with 5 μg of total RNA as input. Ribosomal RNA was removed using the Ribo-Zero rRNA Removal Kit (Epicentre, Madison, WI, USA), followed by RNA fragmentation with fragmentation buffer. cDNA synthesis, end-repair, A-tailing, and adapter ligation were performed in accordance with standard NGS protocols. cDNA fragments ranging from 200 to 300 bp were selected using a 2% low-melting-point agarose gel, followed by 15 cycles of PCR amplification with Phusion DNA Polymerase (New England Biolabs). Metatranscriptomic sequencing was conducted by Shanghai Bio Corporation (Shanghai, China) on a next-generation sequencing platform, employing a 150 bp paired-end (PE150) strategy for all libraries.

### Sequence assembly and candidate viral sequence identification

Clean reads were aligned to the host genome (Anas platyrhynchos) using BWA-MEM (v0.7.17) to eliminate host-derived sequences, with additional screening against the human reference genome (GRCh38) to remove potential human contaminant sequences. High-quality reads were *de novo* assembled into contigs for each sample utilizing MEGAHIT (v1.2.9) with the parameter min-contig-len 500 (Li et al., 2015). Assembled sequences ≥ 2,000 bp were screened for viral sequences using DeepVirFinder (criteria: score > 0.7 and *P < 0.05*), VirSorter2 (v2.2.4), VIBRANT (v1.2.1), and the IMG/VR (Integrated Microbial Genomes/Virus) database (v4) (Ren et al., 2017; Kieft et al., 2020). CheckV (v1.0.1) was employed to estimate sequence completeness by comparison against a database of complete viral genomes; sequences categorized as ‘Not-determined’ were excluded prior to downstream analysis (Kieft et al., 2020).

### Metaviromic vOTU generation

To facilitate both within- and between-sample/group comparative analyses of viral sequences, viral genomes were clustered into viral operational taxonomic units (vOTUs). Candidate viral sequences were compared pairwise utilizing the phageannotator tool. Sequences exhibiting ≥ 95% similarity across ≥ 85% of their length were classified as belonging to the same vOTU, with the longest sequence designated as the representative sequence for that vOTU (Camargo et al., 2024).

### vOTU taxonomic annotation and host prediction

Given the high genomic divergence of viruses, DNA similarity-based methods are often inadequate for taxonomic assignment. Therefore, vOTUs were annotated for taxonomy and host prediction using the geNomad tool (v1.5.0), which employs a gene-centric approach integrating multiple lines of evidence for classification (Camargo et al., 2024). Additionally, protein-level taxonomic annotation was performed by alignment against the NCBI NR database.

### vOTU gene prediction and functional annotation

Genes within vOTUs were predicted using METAProdigal (v2.6.3) (Hyatt et al., 2012). After removal of host sequences, clean reads were aligned back to the non-redundant viral sequence and gene catalogs using BWA-MEM to calculate read counts, abundance, and coverage for each viral sequence or gene. The predicted protein sequences were aligned against the following curated databases to obtain functional annotations: (1) SARG (Structured Antibiotic Resistance Gene database, v3.2) for ARG annotation; (2) BacMet (antibacterial biocide and metal resistance genes database, v2.0) for MRG and BRG annotation; (3) VFDB (Virulence Factor Database, 2022 release) for VF annotation; (4) KEGG (Kyoto Encyclopedia of Genes and Genomes, release 105.0) for metabolic pathway annotation; (5) COG (Clusters of Orthologous Groups, 2020 update) for functional classification; (6) GO (Gene Ontology, 2023 release) for functional annotation; (7) CAZy (Carbohydrate-Active Enzymes database, 2023 release) for enzyme annotation; and (8) Swiss-Prot (2023 release) for high-quality protein annotation. All database searches were conducted using DIAMOND (v2.1.8) BLASTP with an E-value cutoff of 1e^-^^5^.

### Alpha and beta diversity analysis

Rarefaction analysis was conducted using Mothur (v1.48.0) to evaluate viral community diversity indices, including the Richness index, Shannon diversity index, and Simpson diversity index (Schloss et al., 2009). Beta diversity analysis was performed utilizing the vegan package (v2.6-4) in R (v4.3.0). A Bray-Curtis distance matrix was calculated to assess viral community similarity, followed by permutation tests with 999 permutations. Spearman’s rank correlation coefficients between viruses and functional elements were computed using R packages. Significantly correlated pairs (absolute correlation coefficient > 0.6, *P < 0.05*) were selected for further analysis. Correlation heatmaps and network diagrams were visualized using Gephi (v0.10). Principal Component Analysis (PCA), Principal Coordinates Analysis (PCoA), and Non-metric Multidimensional Scaling (NMDS) were executed using the vegan package in R.

### Single-sample in-depth metavirome analysis

Although this analysis is based on a single sample and the results should be interpreted with caution and are not generalizable to the broader population, an exploratory pilot characterization was conducted on one representative sample to characterize the viral functional landscape at higher resolution. This sample was selected from the DL-D (low feed efficiency) group because it exhibited the most distinct viral community profile and the lowest antimicrobial resistance gene (ARG) abundance among the experimental groups, making it a particularly informative candidate for in-depth virome exploration. This analysis encompassed comprehensive taxonomic profiling of viral contigs at the phylum, class, order, family, genus, and species levels using NCBI NR taxonomy, followed by KEGG pathway enrichment analysis with mapping to Level 1, 2, and 3 pathway hierarchies. COG functional category assignment and GO term annotation at levels 2–4 were also performed. Additionally, CAZyme family and class annotation was conducted, along with the integration of ARG, MRG, BRG, and VF annotations at the gene level. Viral genome completeness was assessed using CheckV, with quality tiers assigned following MIUViG (Minimum Information about an Uncultivated Virus Genome) standards (Nayfach et al., 2021; Roux et al., 2019).

### Statistical analysis

Experimental data were analyzed using one-way analysis of variance (ANOVA) with Prism software (GraphPad, v9.0), and results were graphically presented with asterisks denoting significant differences: *P < 0.05, **P < 0.01, ***P < 0.001 (GraphPad Software, 2025). Alpha and beta diversity metrics were calculated after rarefying all samples to a uniform sequencing depth. Feature abundances were normalized based on their relative abundance in each sample, and taxonomic classification was performed using the SILVA classifier (release 138) (Camargo et al., 2023). All diversity metrics for the samples were computed using QIIME2 (v2023.5). Sequence alignment was conducted using BLAST (v2.14.1), and representative sequences were annotated against multiple reference databases as specified above. All statistical analyses were corrected for multiple testing using the Benjamini-Hochberg false discovery rate (FDR) method where applicable.

## Results

### Sequencing output, viral sequence identification, and abundance quantification

A total of approximately 1.01×10–^9^ raw paired-end reads (approximately 151.5 Gb) were generated from the 20 DNA virome samples. After quality filtering using Trimmomatic (removing adapters, trimming low-quality bases, and discarding reads < 75 bp), approximately 9.76×10–^8^ clean reads (approximately 145.6 Gb) were retained, with Q20 and Q30 values exceeding 97.75% and 93.88%, respectively. Following host genome subtraction using BWA-MEM, approximately 4.31×10–^8^ non-host reads were retained for downstream analyses. *De novo* assembly using MEGAHIT yielded a total of 5,170,738 contigs (≥ 500 bp) across all samples. Screening of contigs ≥ 2,000 bp using DeepVirFinder, VirSorter2, VIBRANT, and the IMG/VR database identified 57,032 candidate viral sequences. After quality assessment with CheckV and removal of sequences classified as Not-determined2, 23,130 viral sequences were retained, comprising 80 complete, 78 high-quality, 208 medium-quality, and 22,764 low-quality genomes. Clustering at 95% average nucleotide identity (ANI) over 85% alignment coverage yielded 18,321 vOTUs with a total length of 95.33 Mb, an N50 of 5,970 bp, and a GC content of 52.22%. Viral sequence and gene abundances were quantified using the TPM (Transcripts Per Million) method for gene-level comparisons, while relative abundance (percentage of total mapped reads in each sample) was used for community composition analyses. Alpha and beta diversity metrics were calculated after rarefying all samples to a uniform sequencing depth.

### Compound microbial intelligent membrane fermentation significantly changes the viral communities in duck litter

**Figure 1:**
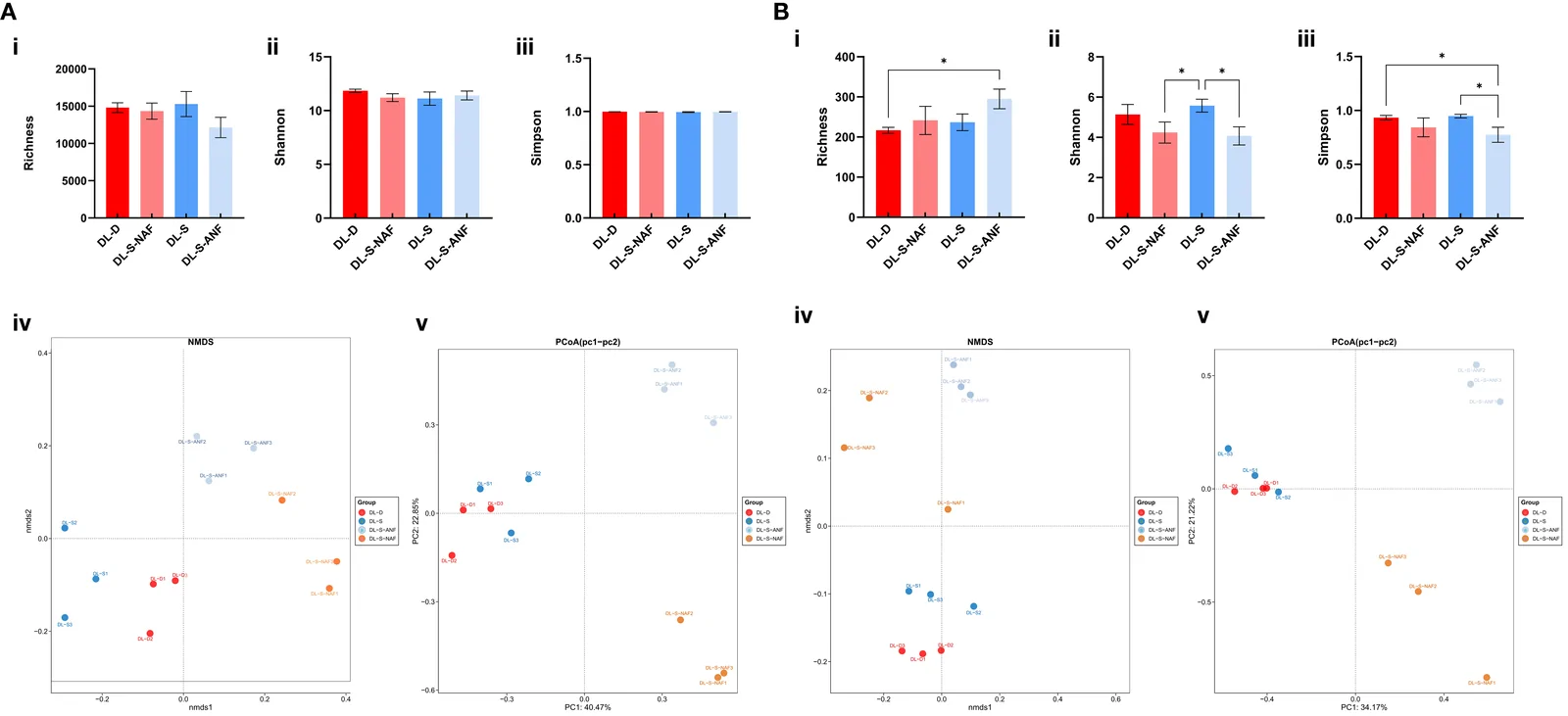
Alpha and Beta Diversity Analysis of the Viral Community in Duck Litter. **(Ai–iii)** DNA viral community alpha diversity indices [**(i)**: Richness; **(ii)**: Shannon; **(iii)**: Simpson]. **(iv, v)** DNA viral community beta diversity analysis [**(iii)**: NMDS; **(iv)**: PCoA, where PC1 and PC2 explained 40.47% and 22.85% of the variance, respectively]. **(Bi–v)** RNA viral community alpha diversity indices [**(i)**: Richness; **(ii)**: Shannon; **(iii)**: Simpson]. **(iv, v)** RNA viral community beta diversity analysis [**(iv)**: NMDS; **(v)**: PCoA, where PC1 and PC2 explained 34.17% and 21.22% of the variance, respectively]. *P < 0.05.

Alpha diversity analysis was conducted to evaluate the species richness and diversity of both DNA and RNA viruses present in the litter samples. For DNA viruses, the DL-D group exhibited decreasing trends in Richness, Shannon, and Simpson indices; however, these differences were not statistically significant, suggesting comparable species richness and diversity across the groups. For RNA viruses, the Richness index also showed a non-significant decreasing trend, indicating similar species richness among the groups. In contrast, analysis of the Shannon and Simpson indices revealed significant differences between the DL-S group and both the DL-S-NAF and DL-S-ANF groups. Furthermore, the Shannon index demonstrated significant differences between the DL-D, DL-S, and DL-S-ANF groups, indicating significant changes in RNA viral community among certain groups (**Figure 1**).

Beta diversity analysis assessed the compositional differences in viral communities among litter samples. Both Non-metric Multidimensional Scaling (NMDS) and Principal Coordinates Analysis (PCoA) revealed significant differences in viral community structure across the experimental groups, as well as notable heterogeneity in species composition among samples within the same groups (Guo et al., 2021). PCoA provided quantitative assessment of these differences: for DNA viruses, principal coordinates PC1 and PC2 accounted for 63.32% of community variation, contributing 40.47% and 22.85%, respectively; for RNA viruses, PC1 and PC2 together explained 55.39% of community variation, with contributions of 34.17% and 21.22%, respectively (**Figure 1**).

### Compound microbial intelligent membrane fermentation significantly alters viral abundance at the phylum level

**Figure 2:**
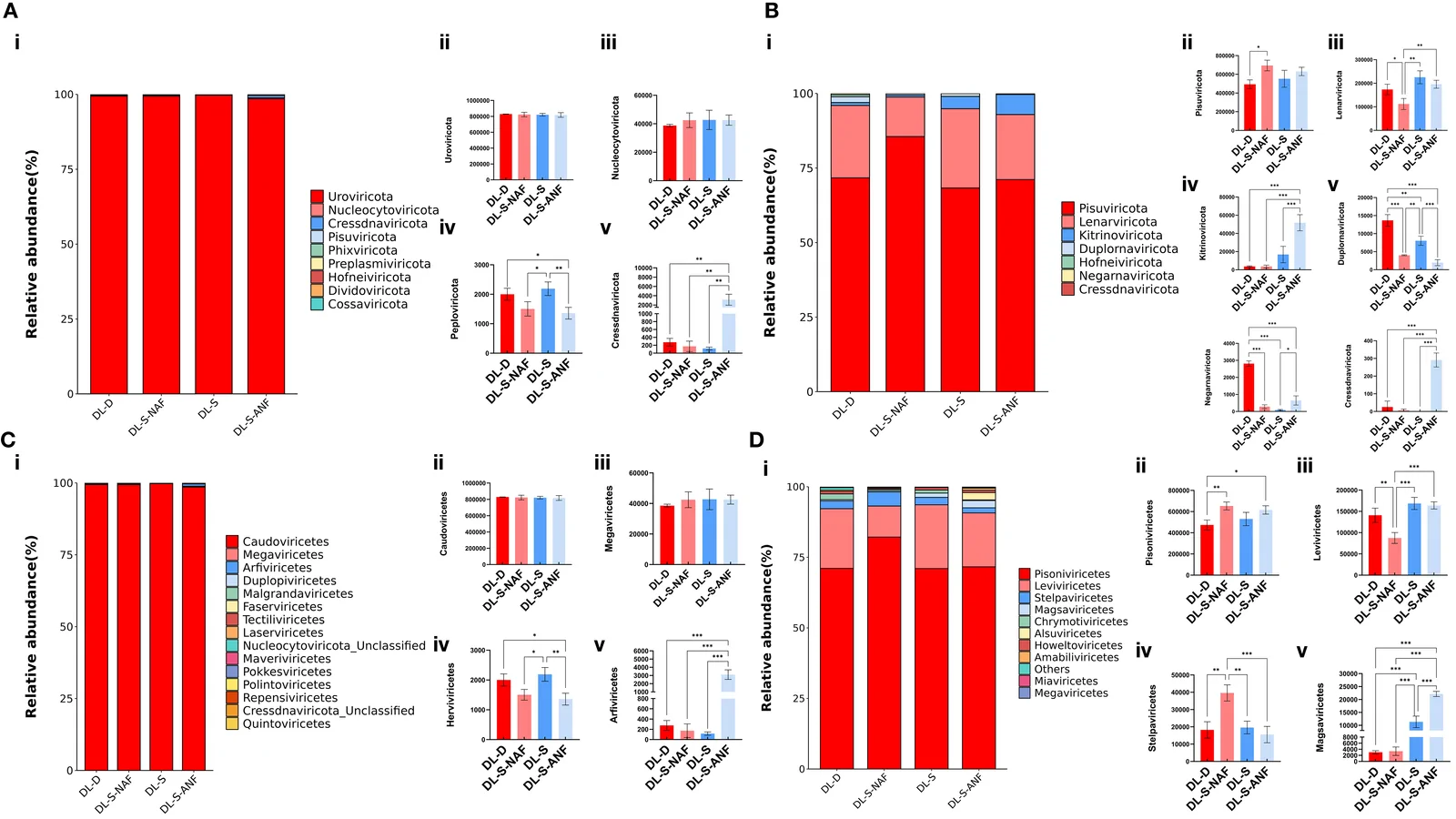
Taxonomic Composition, Relative Abundance, and Intergroup Differences of Viral Communities. **(Ai–v)** DNA virus phylum level: **(i)** community composition; **(ii–v)** intergroup differences for *Uroviricota*, *Nucleocytoviricota*, *Peploviricota*, *Cressdnaviricota*. **(Bi–vii)** RNA virus phylum level: **(Bi)** community composition; **(Bii–vii)** intergroup differences for *Pisuviricota*, *Lenarviricota*, *Kitrinoviricota*, *Duplornaviricota*, *Negarnaviricota*. (**Ci–v)** DNA virus class level: **(Ci)** community composition; **(Cii–v)** intergroup differences for *Caudoviricetes*, *Megaviricetes*, *Herviviricetes*, *Arfiviricetes*. **(Di–v)** RNA virus class level: **(Di)** community composition; **(Dii–v)** intergroup differences for *Pisoniviricetes*, *Leviviricetes*, *Stelpaviricetes*, *Magsaviricetes*. *P < 0.05, **P < 0.01, ***P < 0.001.

At the DNA virus phylum level, *Uroviricota* and *Nucleocytoviricota* were the predominant viral groups across all four sample categories (**Figure 2**). The relative abundances of *Uroviricota* in the DL-D, DL-S-NAF, DL-S, and DL-S-ANF groups were 95.05%, 94.61%, 94.69%, and 94.38%, respectively, while the corresponding abundances of *Nucleocytoviricota* were 4.44%, 4.89%, 4.93%, and 4.92%. The DL-S-ANF group showed the highest abundance of *Cressdnaviricota*. Compared to the DL-S group, a significant downregulation of *Peploviricota* abundance was observed in the DL-S-NAF and DL-S-ANF groups, whereas a significant upregulation of *Cressdnaviricota* abundance was noted in the DL-S-ANF group.

At the RNA virus phylum level, *Pisuviricota* and *Lenarviricota* emerged as the dominant viral phyla. The relative abundances of *Pisuviricota* in the DL-D, DL-S-NAF, DL-S, and DL-S-ANF groups were 71.14%, 85.27%, 68.79%, and 71.53%, respectively, while those of *Lenarviricota* were 25.04%, 13.75%, 28.07%, and 22.19%. *Pisuviricota* was the most abundant in the DL-S-NAF group. *Negarnaviricota* and *Duplornaviricota* exhibited their highest abundances in the DL-D group, and *Kitrinoviricota* was more prevalent in the DL-S and DL-S-ANF groups. When comparing the DL-S-NAF group to the DL-D group, a significant downregulation of *Pisuviricota* abundance was observed, accompanied by a significant upregulation of *Lenarviricota*, *Negarnaviricota*, and *Duplornaviricota*. In contrast, the DL-D group demonstrated significant upregulation of *Negarnaviricota* and *Duplornaviricota* compared to the DL-S group, while the DL-S-NAF group exhibited significant downregulation of *Lenarviricota* and *Duplornaviricota*. In the DL-S-ANF group, the abundances of *Kitrinoviricota*, *Negarnaviricota*, and *Cressdnaviricota* were significantly upregulated, whereas *Duplornaviricota* was significantly downregulated.

### Compound microbial intelligent membrane fermentation significantly alters viral abundance at the class level

At the DNA virus class level, *Caudoviricetes* and *Megaviricetes* were the predominant viral classes across all four sample groups. The relative abundances of *Caudoviricetes* in the DL-D, DL-S-NAF, DL-S, and DL-S-ANF groups were 95.06%, 94.61%, 94.69%, and 94.38%, respectively, while those of *Megaviricetes* were 4.43%, 4.88%, 4.93%, and 4.91%. *Caudoviricetes* exhibited the highest abundance in the DL-D group, whereas *Megaviricetes* peaked in the DL-S group, and *Arfiviricetes* was most abundant in the DL-S-ANF group. The abundance of *Herviviricetes* was significantly downregulated in the DL-S-NAF group compared to the DL-S group. In the DL-S-ANF group, the abundance of *Arfiviricetes* was significantly upregulated, while that of *Herviviricetes* was significantly downregulated.

At the RNA virus class level, *Pisoniviricetes* and *Leviviricetes* were identified as the dominant viral classes. The relative abundances of *Pisoniviricetes* in the DL-D, DL-S-NAF, DL-S, and DL-S-ANF groups were 71.08%, 82.17%, 71.02%, and 71.63%, respectively, whereas those of *Leviviricetes* were 21.15%, 11.00%, 22.59%, and 19.09%. *Pisoniviricetes* was most abundant in the DL-S-NAF group, while *Leviviricetes* and *Stelpaviricetes* reached their peak in the DL-S group, and *Magsaviricetes* was most prevalent in the DL-S-ANF group. In comparison to the DL-S-NAF group, the DL-D group exhibited significant downregulation of *Pisoniviricetes* and *Stelpaviricetes*, while the abundance of *Leviviricetes* was significantly upregulated. Additionally, when comparing the DL-S group to the DL-S-NAF group, significant downregulation of *Leviviricetes* and *Magsaviricetes* was observed, whereas *Stelpaviricetes* showed significant upregulation. In the DL-S-ANF group, *Magsaviricetes* exhibited a significant increase in abundance (**Figure 2**).

### Single-sample in-depth analysis of duck litter reveals metavirome characteristics

The in-depth metavirome analysis of a representative duck litter sample identified a total of 343 viral genes, of which 176 (51.3%) were annotated via the NCBI NR database, 107 (31.2%) via KEGG, 121 (35.3%) via Swiss-Prot, 40 (11.7%) via COG, and 18 (5.3%) via GO (**Supplementary Table 1**). A total of 210 genes (61.2%) received functional annotation in at least one database. Viral taxonomic profiling at the NR level revealed that the viral community was dominated by the phylum *Uroviricota* (class *Caudoviricetes*), including members of the families *Winoviridae*, *Salasmaviridae*, *Mesyanzhinovviridae*, and *Orlajensenviridae*, with *Caudoviricetes* sp., *Peternellavirus* peternella, and *Crassvirales* sp. as the most abundant taxa. The phylum *Nucleocytoviricota* (class *Megaviricetes*) was also well-represented, with *Mimiviridae* sp. ChoanoV1, *Homavirus* sp., and *Mimivirus* reunion among the dominant members (**Supplementary Figure 1**).

KEGG pathway enrichment analysis exhibited that viral genes were predominantly associated with Metabolism (63,610 RPKM), followed by Genetic Information Processing (129,783 RPKM), with translation-related functions (99,441 RPKM) constituting the largest category. Within metabolism, carbohydrate metabolism (17,449 RPKM), energy metabolism (11,629 RPKM), and metabolism of cofactors and vitamins (17,785 RPKM) were the most highly represented categories. Key enriched pathways included carbon metabolism (ko01200), biosynthesis of cofactors (ko01240), and glycolysis/gluconeogenesis (ko00010; **Supplementary Figure 2**).

The viral resistome analysis in the representative sample identified multiple ARG subtypes, including TaeA (efflux pump, pleuromutilin/tiamulin resistance), *Bado_rpoB_RIF* (rifamycin resistance), and aminocoumarin resistance genes. Among MRGs, genes conferring resistance to arsenic (arsT), mercury (merA), chromium (ruvB), copper/zinc (actA), and nickel/cobalt (fecE) were detected. BRG annotation revealed genes associated with resistance to quaternary ammonium compounds (QACs), triclosan, hydrogen peroxide, and hydrochloric acid. VF annotation identified genes involved in effector delivery systems (ricA, clpV), immune modulation (glycosyltransferases), biofilm formation (algI, mucP), exoenzyme production (eno), and stress survival (katA; **Supplementary Table 2**).

### Compound microbial intelligent membrane fermentation significantly decreases antibiotic resistance gene loads

**Figure 3:**
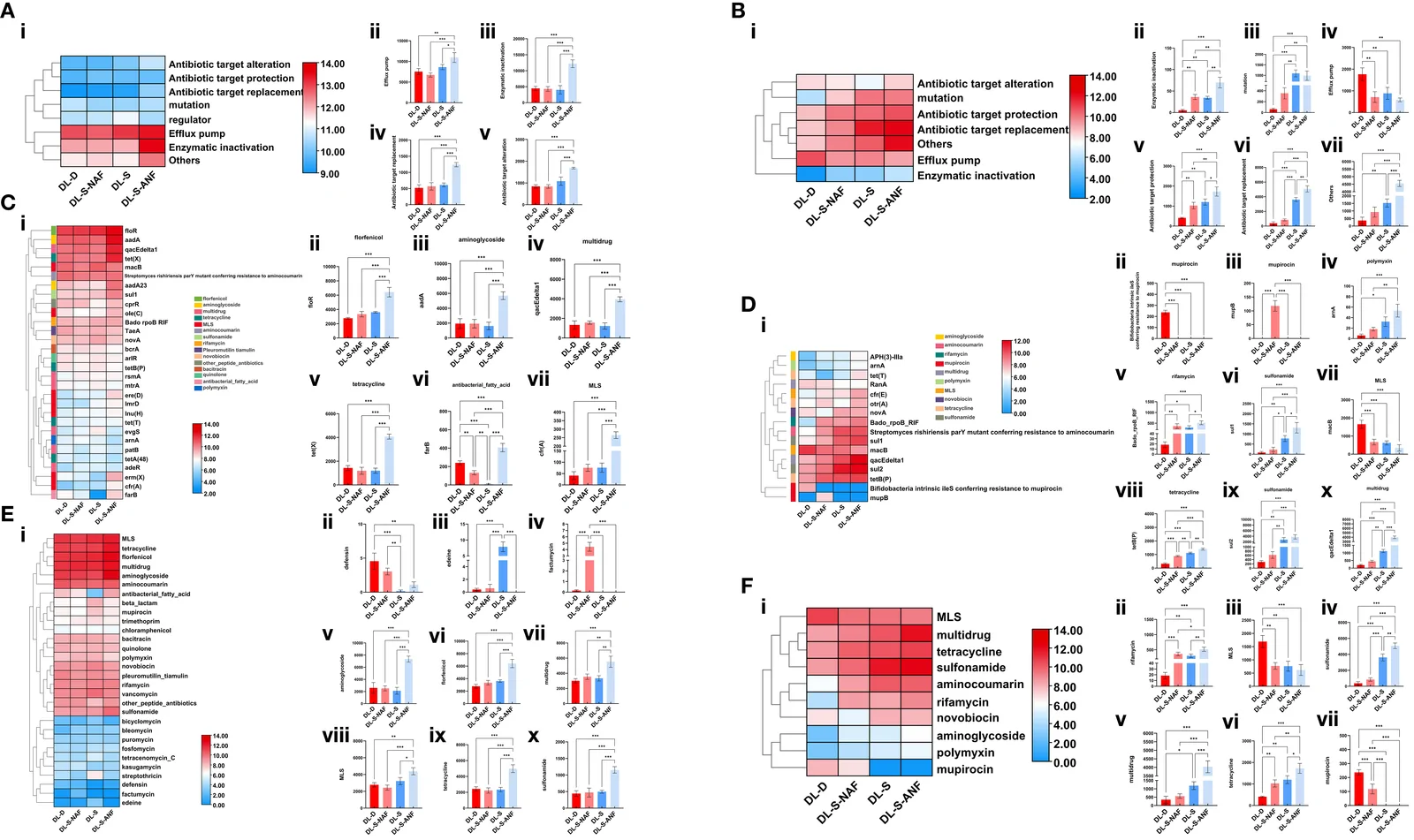
Heatmaps and Intergroup Difference Analysis of Antibiotic Resistance Genes Abundance (RPKM). **(Ai–v)** DNA virus ARG mechanisms; **(Bi–vii)** RNA virus ARG mechanisms; **(Ci–vii)** DNA virus ARG subtypes; **(Di–x)** RNA virus ARG subtypes; **(Ei–x)** DNA virus-targeted antibiotics; **(Fi–vii)** RNA virus-targeted antibiotics. *P < 0.05, **P < 0.01, ***P < 0.001. See text for detailed statistical comparisons.

The abundances of efflux pumps, enzymatic inactivation, antibiotic target replacement, and antibiotic target alteration in the DL-D, DL-S-NAF, and DL-S groups (**Figure 3A**, DNA virus) were significantly lower than those in the DL-S-ANF group (*P < 0.05*).

In the heatmap of RNA virus antibiotic resistance mechanisms (**Figure 3B**), the abundances of enzymatic inactivation, mutation, antibiotic target protection, antibiotic target replacement, and other mechanisms in the DL-D group were significantly lower than those in the DL-S and DL-S-ANF groups (*P < 0.01*). Specifically, the abundances of enzymatic inactivation and antibiotic target protection were significantly lower in the DL-D group compared to the other three groups (*P < 0.01*), while the abundance of efflux pump mechanisms was significantly higher in the DL-D group than in the other three groups (*P < 0.01*)—a notable outlier addressed in the Discussion. In the DL-S-NAF group, the abundances of mutation and antibiotic target replacement were significantly lower than those in the DL-S group (*P < 0.01*), and the abundances of enzymatic inactivation, mutation, antibiotic target protection, antibiotic target replacement, and other mechanisms were significantly lower than those in the DL-S-ANF group (*P < 0.01*). In the DL-S group, the abundances of enzymatic inactivation, antibiotic target protection, antibiotic target replacement, and other mechanisms were significantly lower than those in the DL-S-ANF group (*P < 0.05*).

Antibiotic resistance gene of regarding DNA virus (**Figure 3C**), the abundances of *floR*, *aadA*, *qacE delta1*, *tet(X)*, and *cfr(A)* in the DL-D, DL-S-NAF, and DL-S groups were significantly lower compared to those in the DL-S-ANF group (*P < 0.001*). The abundance of *farB* in the DL-D group was significantly higher than that in the DL-S-NAF and DL-S groups, yet significantly lower than that in the DL-S-ANF group (*P < 0.01*). In the DL-S group, the abundance of *farB* was significantly lower than that in the DL-S-ANF group (*P < 0.001*).

Turning to RNA virus ARG subtypes (**Figure 3D**) *arnA*, *Bado_rpoB_RIF*, *sul1*, *tetB(P)*, *sul2*, and *qacEdelta1* in the DL-D group were significantly lower than those in the DL-S and DL-S-ANF groups (*P < 0.05*). Furthermore, the abundances of *Bado_rpoB_RIF* and *tetB(P)* were significantly lower compared to the DL-S-NAF group (*P < 0.01*). Conversely, the abundances of Bifidobacteria intrinsic ileS (conferring resistance to mupirocin) and macB were significantly higher than those in the other three groups (*P < 0.001*). In the DL-S-NAF group, the abundances of *sul1*, *tetB(P)*, *sul2*, and *qacEdelta1* were significantly lower than those in the DL-S and DL-S-ANF groups (*P < 0.01*), while the abundance of *arnA* was significantly lower than that in the DL-S-ANF group (*P < 0.01*). In contrast, the abundance of *mupB* in the DL-S-NAF group was significantly higher than that in the other three groups (*P < 0.001*). In the DL-S group, the abundances of *Bado_rpoB_RIF*, *sul1*, *tetB(P)*, and *qacEdelta1* were significantly lower than those in the DL-S-ANF group (*P < 0.05*).

With respect to antibiotic class-level profiling (**Figure 3E**) aminoglycoside, florfenicol, multidrug, MLS, tetracycline, and sulfonamide in the DL-D, DL-S-NAF, and DL-S groups were significantly lower than those in the DL-S-ANF group (*P < 0.05*). Furthermore, the abundance of defensin in the DL-D group was significantly higher than that in the DL-S and DL-S-ANF groups (*P < 0.01*). In the DL-S-NAF group, defensin abundance was significantly elevated compared to the DL-S group (*P < 0.01*), and factumycin abundance was significantly higher than in the other three groups (*P < 0.001*). In the DL-S group, the abundance of edeine was significantly greater than that in the other three groups (*P < 0.001*).

A parallel analysis of RNA virus antibiotic class distribution revealed that rifamycin, sulfonamide, multidrug, and tetracycline in the DL-D group was significantly lower compared to the DL-S and DL-S-ANF groups (*P < 0.05*). Additionally, the abundances of rifamycin and tetracycline were significantly lower than those in the DL-S-NAF group (*P < 0.01*). Conversely, the abundances of MLS and mupirocin were significantly higher than those in the other three groups (*P < 0.01*). In the DL-S-NAF group, the abundances of rifamycin, sulfonamide, multidrug, and tetracycline were significantly lower than those in the DL-S-ANF group (*P < 0.05*), with sulfonamide abundance also significantly lower than that in the DL-S group (*P < 0.05*). Conversely, mupirocin abundance in the DL-S-NAF group was significantly higher than that in the DL-S and DL-S-ANF groups (*P < 0.001*). In the DL-S group, the abundances of rifamycin, sulfonamide, multidrug, and tetracycline were significantly lower than those in the DL-S-ANF group (*P < 0.05*).

### Compound microbial intelligent membrane fermentation significantly reduces metal resistance genes

**Figure 4:**
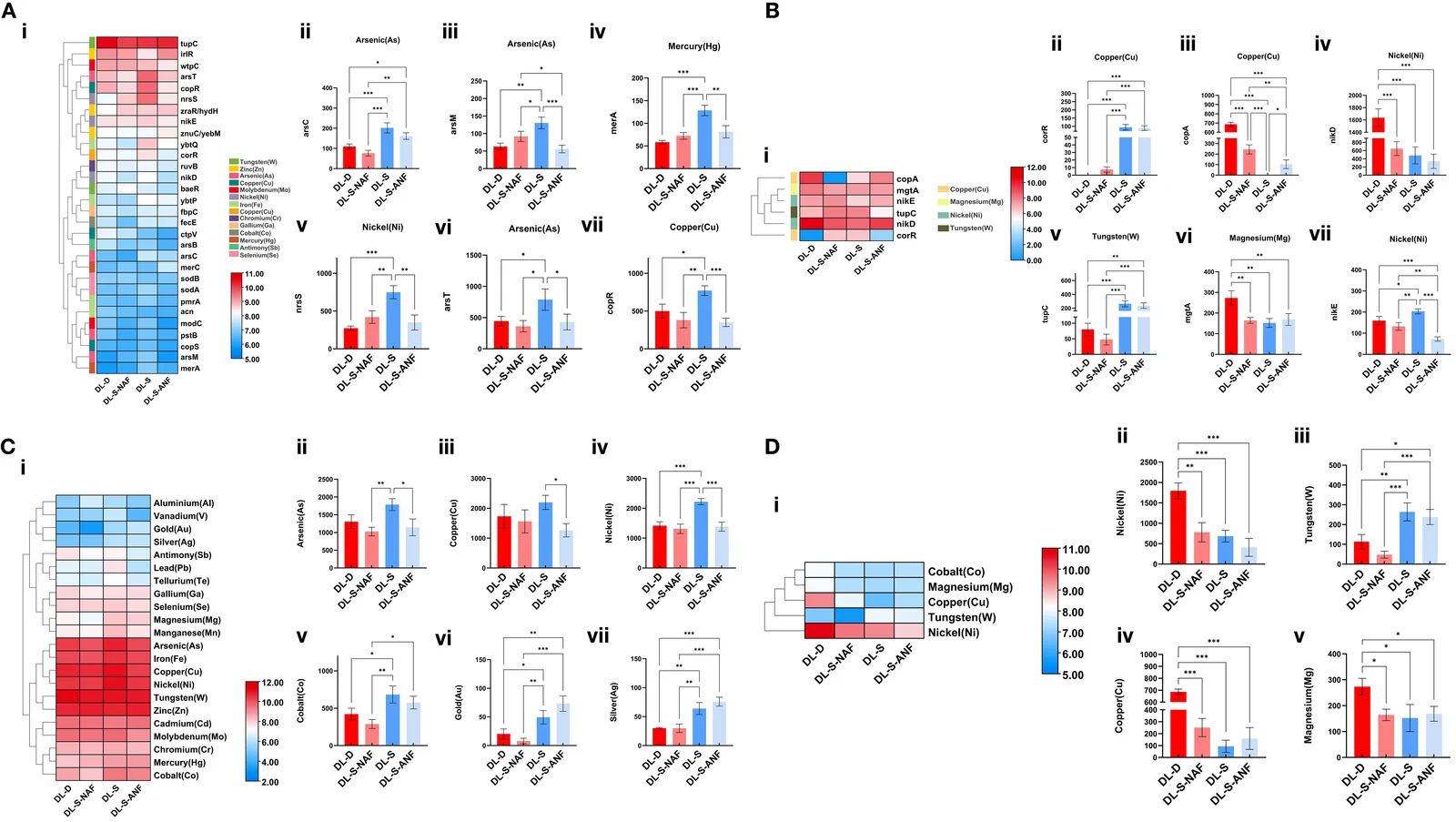
Heatmaps and Intergroup Difference Analysis of Metal Resistance Genes Abundance (RPKM). **(Ai–vii)** DNA virus MRG subtypes; **(Bi–vii)** RNA virus MRG subtypes; **(Ci–vii)** DNA virus-associated metals; **(Di–v)** RNA virus-associated metals. *P < 0.05, **P < 0.01, ***P < 0.001. See text for detailed statistical comparisons.

The abundance of metal resistance genes (*MRGs*) in DNA virus exhibited the following trend: DL-D < DL-S-NAF < DL-S-ANF < DL-S. As illustrated in the heatmap of DNA virus MRG subtypes. (**Figure 4A**), the abundances of *arsC*, *arsM*, *merA*, *nrsS*, *arsT*, and *copR* in the DL-D group were significantly lower than those in the DL-S group (*P < 0.05*). Specifically, the abundance of *arsC* was significantly lower in the DL-D group compared to the DL-S-ANF group (*P < 0.05*). In the DL-S-NAF group, the abundances of *arsC*, *arsM*, *merA*, *nrsS*, *arsT*, and *copR* were significantly lower than those in the DL-S group (*P < 0.05*). Furthermore, the abundance of *arsC* was significantly lower than that in the DL-S-ANF group (*P < 0.05*), whereas the abundance of *arsM* was significantly higher than that in the DL-S-ANF group (*P < 0.05*). In the DL-S group, the abundances of *arsM*, *merA*, *nrsS*, *arsT*, and *copR* were significantly higher than those in the other three groups (*P < 0.05*).

In the heatmap depicting RNA virus MRG subtypes (**Figure 4B**), the abundances of *corR* and *tupC* in the DL-D group were significantly lower than those in the DL-S and DL-S-ANF groups (*P < 0.01*), while the abundance of *nikE* was lower compared to the DL-S group (*P < 0.05*). Conversely, the abundances of *copA*, *nikD*, and *mgtA* in the DL-D group were significantly higher than those in the other three groups (*P < 0.01*), and the abundance of *nikE* was greater than that in the DL-S-ANF group (*P < 0.05*). In the DL-S-NAF group, the abundances of *corR*, *tupC*, and *nikE* were lower than those in the DL-S group (*P < 0.01*), and the abundances of *corR* and *tupC* were also lower than those in the DL-S-ANF group (*P < 0.001*). Notably, the abundance of *copA* was higher than that in the DL-S group (*P < 0.001*), and the abundances of both *copA* and *nikE* exceeded those in the DL-S-ANF group (*P < 0.01*).

As illustrated in the heatmap of DNA virus-associated metals (**Figure 4C**), the abundances of nickel (Ni), cobalt (Co), gold (Au), and silver (Ag) in the DL-D group were significantly lower than those in the DL-S group (*P < 0.05*). Furthermore, the abundances of gold (Au) and silver (Ag) in the DL-D group were markedly lower than those in the DL-S-ANF group (*P < 0.01*). In the DL-S-NAF group, the abundances of arsenic (As), nickel (Ni), cobalt (Co), gold (Au), and silver (Ag) were also significantly lower than in the DL-S group (*P < 0.01*), with cobalt (Co), gold (Au), and silver (Ag) showing lower abundances compared to the DL-S-ANF group (*P < 0.05*). Conversely, in the DL-S group, the abundances of arsenic (As), copper (Cu), and nickel (Ni) were found to be higher than those in the DL-S-ANF group (*P < 0.05*).

In the heatmap of RNA virus-associated metals (**Figure 4D**), the abundances of nickel (Ni), copper (Cu), and magnesium (Mg) in the DL-D group were significantly higher than those in the other three groups (*P < 0.05*). Conversely, the abundance of tungsten (W) was lower in the DL-D group compared to the DL-S and DL-S-ANF groups (*P < 0.05*). Additionally, in the DL-S-NAF group, the abundance of tungsten (W) was significantly lower than that in the DL-S and DL-S-ANF groups (*P < 0.001*).

### Biocide resistance genes are decreased by compound microbial intelligent membrane fermentation

**Figure 5:**
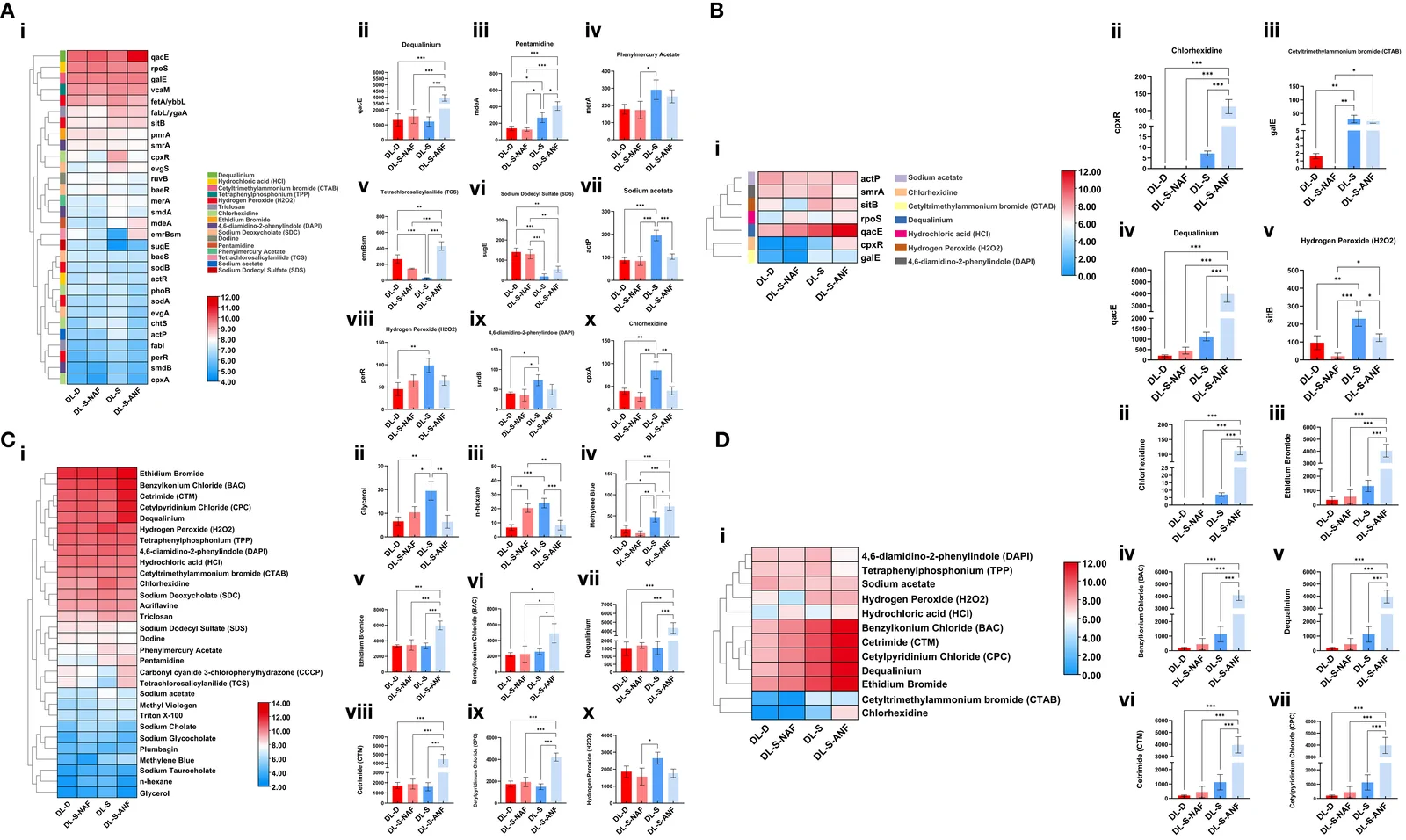
Heatmaps and Intergroup Difference Analysis of Biocide Resistance Genes Abundance (RPKM). **(Ai–x)** DNA virus BRG subtypes; **(Bi–v)** RNA virus BRG subtypes; **(Ci–x)** DNA virus-associated biocides; **(Di–vii)** RNA virus-associated biocides. *P < 0.05, **P < 0.01, ***P < 0.001. See text for detailed statistical comparisons.

The distribution of biocide resistance genes (BRGs) in DNA virus exhibited the following trend: DL-D < DL-S-NAF < DL-S ≈ DL-S-ANF. (**Figure 5D**), the abundances of *mdeA*, *actP*, *perR*, *smdB*, and *cpxA* in the DL-D group were significantly lower than those in the DL-S group (*P < 0.05*). Additionally, the abundances of *qacE*, *mdeA*, and *emrBsm* were significantly lower than those in the DL-S-ANF group (*P < 0.05*), whereas the abundance of *sugE* was higher than that in the DL-S-ANF group (*P < 0.01*). In the DL-S-NAF group, the abundances of *mdeA*, *merA*, *actP*, *smdB*, and *cpxA* were significantly lower than those in the DL-S group (*P < 0.05*). The abundance of *sugE* was higher than that in the DL-S group (*P < 0.001*), while the abundances of *qacE*, *mdeA*, and *emrBsm* were lower than those in the DL-S-ANF group (*P < 0.001*). Conversely, the abundance of *sugE* was higher than that in the DL-S-ANF group (*P < 0.01*).

The abundances of *galE* and *sitB* in the DL-D group were significantly lower than those in the DL-S group (**Figure 5B**, DNA virus), (*P < 0.01*). Moreover, the abundances of *cpxR* and *qacE* were significantly lower compared to the DL-S-ANF group (*P < 0.001*). In the DL-S-NAF group, the abundances of *galE* and *sitB* were also significantly lower than those in the DL-S group (*P < 0.01*), and the abundances of *cpxR*, *galE*, *qacE*, and *sitB* were significantly lower than those in the DL-S-ANF group (*P < 0.05*).

The abundances of methylene blue, ethidium bromide, benzalkonium chloride (BAC), dequalinium, cetrimide (CTM), and cetylpyridinium chloride (CPC) in the DL-S-ANF group were significantly higher compared to the other three groups (*P < 0.05*)(**Figure 5C**, DNA virus).

As shown in the heatmap of RNA virus-associated biocides (**Figure 5D**), the abundances of chlorhexidine, ethidium bromide, benzalkonium chloride (BAC), dequalinium, cetrimide (CTM), and cetylpyridinium chloride (CPC) in the DL-D, DL-S-NAF, and DL-S groups were significantly higher than those in the DL-S-ANF group (*P < 0.001*).

### Virulence factor genes are significantly reduced by compound microbial intelligent membrane fermentation

**Figure 6:**
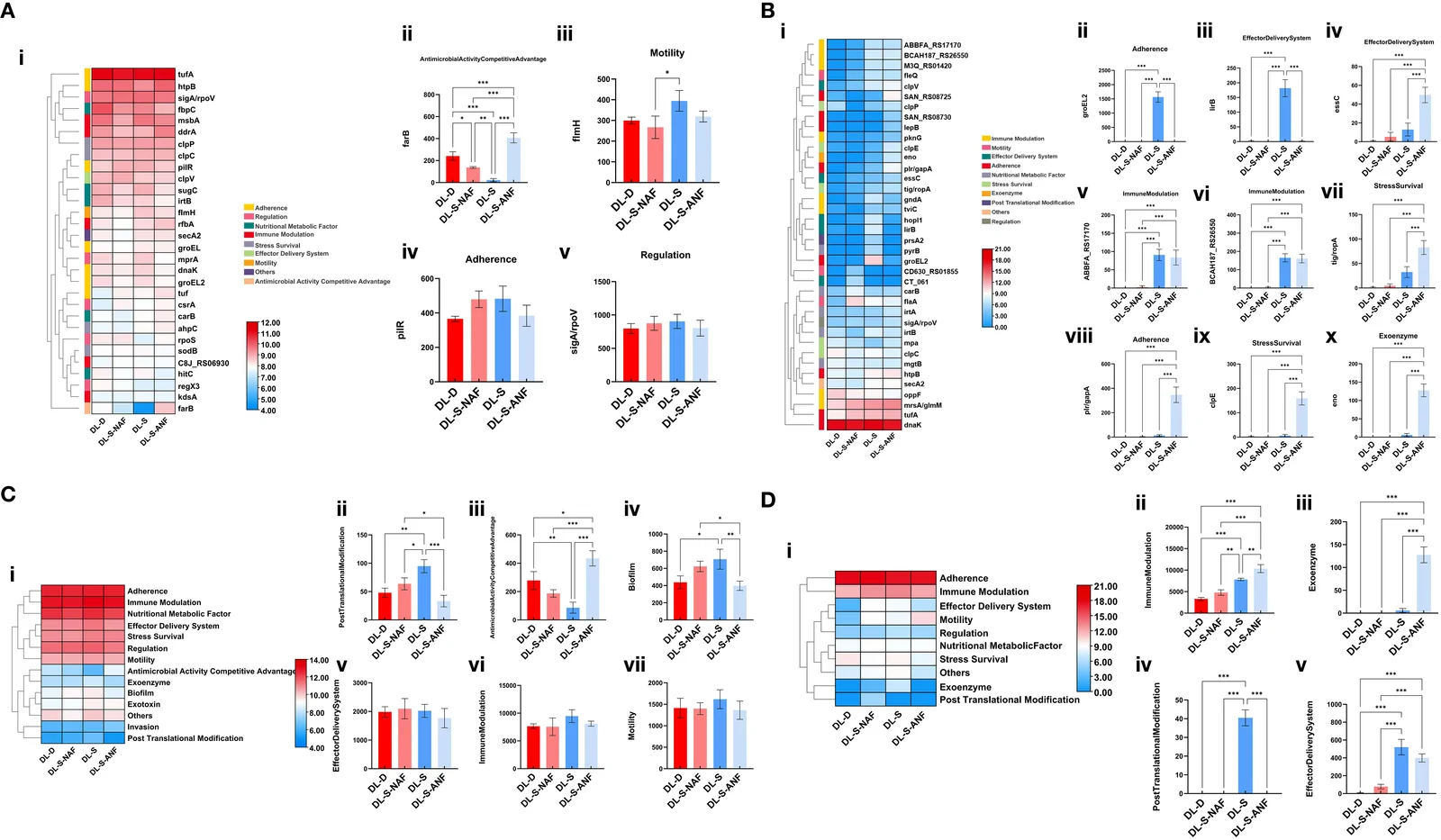
Heatmaps and Intergroup Difference Analysis of Virulence Factor Genes Abundance (RPKM). **(Ai–v)** DNA virus VF gene subtypes; **(Bi–x)** RNA virus VF gene subtypes; **(Ci–vii)** DNA virus VF functions; **(Di–v)** RNA virus VF functions. *P < 0.05, **P < 0.01, ***P < 0.001. See text for detailed statistical comparisons.

The abundance of virulence factor (VF) genes in DNA virus exhibited a hierarchical trend: DL-D < DL-S-NAF < DL-S < DL-S-ANF. As illustrated in the heatmap of DNA virus VF gene subtypes. (**Figure 6A**), the abundance of *farB* in the DL-D group was significantly lower than that in the DL-S-ANF group (*P < 0.001*) but higher than that in the DL-S-NAF and DL-S groups (*P < 0.05*). In the DL-S-NAF group, the abundance of *flmH* was lower than that in the DL-S group (*P < 0.05*), whereas the abundance of *farB* was lower than that in the DL-S-ANF group (*P < 0.001*) but higher than that in the DL-S group (*P < 0.01*).

The heatmap of RNA virus VF gene subtypes (**Figure 6B**) revealed that the abundances of*essC*, *ABBFA_RS17170*, *BCAH187_RS26550*, *tig/ropA*, *plr/gapA*, *clpE*, and *eno* in the DL-D group were significantly lower than those in the DL-S-ANF group (*P < 0.001*). The abundances of *groEL2*, *lirB*, *ABBFA_RS17170*, and *BCAH187_RS26550* were significantly lower than those in the DL-S group (*P < 0.001*). In the DL-S-NAF group, the abundances of *groEL2*, *lirB*, *ABBFA_RS17170*, and *BCAH187_RS26550* were significantly lower than those in the DL-S group (*P < 0.001*), while the abundances of *essC*, *ABBFA_RS17170*, *BCAH187_RS26550*, *tig/ropA*, *plr/gapA*, *clpE*, and *eno* were significantly lower than those in the DL-S-ANF group (*P < 0.001*).

As shown in **Figure 6C**, the abundance of antimicrobial activity/competitive advantage of DNA virus in the DL-D group was significantly lower than that in the DL-S-ANF group (*P < 0.05*). Additionally, the abundances of post-translational modification and biofilm were also found to be lower than those in the DL-S group (*P < 0.05*). In the DL-S-NAF group, the abundance of post-translational modification was lower than in the DL-S group (*P < 0.05*), and the abundance of antimicrobial activity/competitive advantage was lower than in the DL-S-ANF group (*P < 0.001*).

The heatmap presented in **Figure 6D** illustrates the functions of RNA virus virulence factors. Notably, the abundances of immune modulation, post-translational modification, and effector delivery system in the DL-D group were significantly lower than those in the DL-S group (*P < 0.001*). Furthermore, the abundances of immune modulation, exoenzyme, and effector delivery system in the DL-D group were also lower than those in the DL-S-ANF group (*P < 0.001*). In the DL-S-NAF group, the abundances of immune modulation, post-translational modification, and effector delivery system were significantly lower than those in the DL-S group (*P < 0.01*), and the abundances of immune modulation, exoenzyme, and effector delivery system were again lower than in the DL-S-ANF group (*P < 0.01*).

## Discussion

This study employed metaviromics to systematically analyze the viral community structure and resistance gene profiles in duck litter across four different treatments. The comprehensive analysis of viral communities, *ARGs*, *MRGs, BRGs*, and *VFs* provides an integrated perspective on how litter treatment shapes the litter microbiome and resistome. We found that both deep and shallow fermentation significantly reduce viral abundance, particularly that of RNA viruses. The overall abundance of resistance genes follows a clear gradient of DL-D < DL-S-NAF < DL-S < DL-S-ANF. The use of antibiotics in duck farming significantly elevates the co-enrichment of *BRGs* and *VFs*, while the fermentation of duck litter can effectively mitigate this risk. Mechanistically, the sustained high temperatures (80–85°C) drive thermal inactivation of viral particles and degradation of extracellular DNA (Yang et al., 2023), while competitive exclusion of enteric ARG-harboring bacteria by thermophilic microbiota (Youngquist et al., 2016) and organic-acid-mediated disruption of viral envelopes synergistically contribute to the observed reductions (Zhou et al., 2019).

We found no significant differences in the alpha diversity of DNA viral communities among the groups. At the phylum level, *Uroviricota* and *Nucleocytoviricota* were the overwhelmingly dominant taxa, indicating high structural stability of DNA viral communities, particularly bacteriophages, in the litter environment. This finding aligns with the general pattern of phage dominance observed across diverse environments; however, it appears particularly pronounced in the specific niche of duck litter (Chevallereau et al., 2022). The in-depth single-sample analysis corroborated these findings, revealing a diverse assemblage of *Caudoviricetes* (including families *Winoviridae*, *Salasmaviridae*, *Mesyanzhinovviridae*, and *Orlajensenviridae*) and *Megaviricetes* (*Mimiviridae*), consistent with the bacteriophage-dominated viral ecology typically observed in fecal-rich environments. The detection of Riemerella phage vB_RanS_CRP19, a bacteriophage targeting *Riemerella anatipestifer* — a major duck pathogen — suggests that the litter virome may harbor phages with potential biocontrol applications. The structural resilience of DNA viral communities likely reflects the thermostability of double-stranded DNA phage capsids and lysogenic life-cycle adoption under thermal stress, enabling vertical transmission within surviving hosts.

In contrast, RNA viral communities exhibited greater sensitivity to the treatments. Analyses of the Shannon and Simpson indices revealed significant differences between the DL-S group and the others, with the relative abundances of *Pisuviricota* and *Lenarviricota* fluctuating markedly among treatments. *Pisuviricota* was most abundant in the DL-S-NAF group, whereas *Negarnaviricota* and *Duplornaviricota* were significantly enriched in the DL-D group. These differences may be attributed to the distribution of potential duck pathogens. The phylum *Pisuviricota* encompasses significant duck pathogens, including Duck Tembusu virus (TMUV). Its highest abundance in the DL-S-NAF group may be associated with sustained high temperatures and acidic conditions during fermentation, potentially inhibiting certain bacteria while providing a competitive advantage to thermotolerant or acidophilic eukaryotes and their associated viruses (Zhang et al., 2017). The notable enrichment of *Negarnaviricota*, which includes critical pathogens such as influenza viruses, in the DL-D group indicates that the deep anaerobic fermentation environment may be more conducive to the persistence of such viruses. At the class level, the highest abundance of *Pisoniviricetes* in the DL-S-NAF group suggests that deep fermentation (DL-D) may create a more complex, stratified microenvironment: the surface layer provides thermophilic aerobic conditions for manure decomposition, while deeper layers establish stable anaerobic niches that serve as refuges for certain anaerobic microorganisms and their associated viruses (Yang et al., 2023). The heightened sensitivity of RNA viruses is mechanistically attributable to the chemical lability of RNA under the alkaline pH generated by ammonia release during fermentation, coupled with lipolytic disruption of viral envelopes at elevated temperatures (Zhou et al., 2019).

The overall abundance trends of resistance genes clearly indicate two primary driving factors: antibiotic usage is the principal driver of ARG enrichment, while the fermentation process is an effective method for their reduction (Youngquist et al., 2016). The total ARG abundance followed the order DL-D < DL-S-NAF < DL-S < DL-S-ANF. This observation is consistent with findings by Zhou et al., who reported high abundances of the sulfonamide resistance gene*sul1* and the highest total ARG abundance in soil from duck-fish integrated farms in Guangdong, confirming that duck farming environments are significant reservoirs of *ARGs* (Zhou et al., 2019). Moreover, the elevated abundance of *tet(X)* identified in the DL-S-ANF group aligns with the whole-genome study of *R. anatipestifer* (RA) conducted by Sichuan Agricultural University, which reported a*tet(X)* carriage rate of 90.64% in RA isolates, further confirming the widespread dissemination of tetracycline resistance genes in duck farming environments (Zhu et al., 2018). The progressive *ARG* reduction can be attributed to thermal elimination of enteric ARG hosts, suppression of horizontal gene transfer through degradation of mobile genetic elements, and loss of plasmid fitness advantage in the absence of antibiotic selective pressure (Youngquist et al., 2016).

A counterintuitive finding was the relatively high abundance of efflux pump mechanism genes (such as TaeA) in the DL-D group. The single-sample in-depth analysis also detected efflux pump *ARGs* (TaeA, conferring pleuromutilin/tiamulin resistance) and an additional efflux pump BRG (vcaM, associated with QAC and ethidium bromide resistance). This phenomenon may be attributed to adaptive mechanisms activated by microorganisms in response to metabolic stress — such as organic acid accumulation — within the fermentation environment. This observation aligns with the characteristics of multidrug efflux pump genes identified in *R. anatipestifer* (Chen et al., 2018), suggesting that microorganisms may upregulate efflux pump genes even in fermentation litter devoid of antibiotic pressure as a general stress response mechanism. Alternatively, these efflux pumps may serve dual physiological functions, such as metabolite export or pH homeostasis, rather than solely mediating antimicrobial resistance. This is consistent with the substrate promiscuity of MFS and RND-family efflux transporters fulfilling ancestral roles in metabolite export and pH homeostasis under acidic fermentation conditions (Chen et al., 2018), rather than mediating antibiotic resistance perse.

Regarding MRGs, the overall abundance trend was DL-D < DL-S-NAF < DL-S-ANF < DL-S. The significant enrichment of various MRGs (such as *arsM*, *merA*, nrsS) in the DL-S group may be associated with enhanced metal anaerobic reduction in shallow, unfermented litter, leading to increased concentrations of bioavailable metal ions (such as arsenic, mercury). Zhou et al. highlighted the prevalence of heavy metal pollution in farming environments in their study on goose astrovirus (Zhou et al., 2019). Our study further elucidates the selective enrichment effect of litter treatment practices on metal resistance genes. The single-sample analysis identified MRGs for eight metals (Cr, Cu, Zn, As, Hg, Ni, Co, W), demonstrating the broad-spectrum metal resistance potential harbored within the litter virome. The opposing MRG trends between unfermented and fermented groups likely reflect redox-driven metal speciation shifts: oxidative dissolution in aerated shallow litter mobilizes bioavailable metals that select for MRG maintenance, whereas sulfidogenic conditions during thermophilic fermentation promote metal precipitation, alleviating this selective pressure.

The overall trend for BRGs was DL-D < DL-S-NAF < DL-S ≈ DL-S-ANF, while for VFs it was DL-D < DL-S-NAF < DL-S < DL-S-ANF. This study identified significant co-enrichment of multiple BRGs (qacE, *emrBsm*, mdeA) and VFs (essC, *clpE*, *tig/ropA*) within the DL-S-ANF group. This aligns with findings from studies on duck pathogens: a study of *R. anatipestifer* isolates from Guizhou province reported that 16 of 23 RA strains harbored quaternary ammonium compound disinfectant resistance genes (*qacE, qacEdelta1*), while also demonstrating severe multidrug resistance (up to 15 antibiotics) and carrying various resistance genes *(tet(X)*, *ermF, floR*) (Gu et al., 2023). The co-enrichment of *BRGs* and *VFs* in the DL-S-ANF group in our study provides further evidence for the prevalent co-occurrence of disinfectant resistance, antibiotic resistance, and virulence factors in duck farming environments, with their distribution being significantly influenced by treatment practices. This co-enrichment is mechanistically driven by physical linkage of BRG, ARG, and VF determinants on class 1 integrons and conjugative plasmids (Gu et al., 2023), enabling antibiotic-mediated co-selection to amplify resistance and virulence traits through genetic hitchhiking.

The single-sample functional annotation revealed that *VFs* in the duck litter virome span multiple functional categories—including effector delivery systems (*ricA, clpV*), immune modulation (four distinct glycosyltransferases), biofilm formation (algI, mucP), exoenzyme production (eno), exotoxin synthesis (cylG), and stress survival (*katA*) — indicating a broad pathogenic potential encoded within the viral metagenome. The VF functional category profile showed dominance of immune modulation and nutritional/metabolic factors, suggesting that the viral community may contribute to pathogen fitness through multiple virulence mechanisms. Phage-encoded glycosyltransferases may alter host lipopolysaccharide to modulate immune recognition, while biofilm-promoting genes (*algI, mucP*) and the catalase gene katA likely enhance bacterial survival during the oxidative and thermal stresses of the fermentation process.

The significant increase in *BRGs* and *VFs* within the DL-S-ANF group may be driven by co-selection pressure induced by antibiotic use. A pan-genomic assessment of *R. anatipestifer* revealed that over 90% of isolates harbored *tet (X)*, with strains exhibiting high resistance rates to sulfonamides and quinolones (Chen et al., 2018; Zhu et al., 2018). This widespread antibiotic resistance background may facilitate the co-selection and dissemination of *BRGs* and *VFs* through mobile genetic elements, highlighting the urgent need for antibiotic-free farming practices. This co-selection operates through co-resistance (physical linkage on mobile elements), cross-resistance (shared efflux pumps for antibiotics and biocides), and co-regulation (antibiotic-triggered stress regulons upregulating multiple resistance and virulence phenotypes) (Hassan et al., 2026).

This study has several limitations. First, the sample size (n = 5 per group) was relatively modest, which may limit the statistical power to detect subtle differences in viral community composition. Second, the cross-sectional design provides a snapshot view of the virome and resistome; longitudinal studies are needed to understand the temporal dynamics of these microbial communities during fermentation. Third, while metaviromic approaches provide comprehensive sequence-level characterization, functional validation through culture-based or *in vitro* experiments is warranted to confirm the biological significance of the identified resistance genes and virulence factors. Fourth, the study was conducted in a single geographic region (Fujian Province), and the generalizability of these findings to other climatic zones and farming systems requires further investigation. The mechanistic interpretations advanced herein, including thermal inactivation kinetics, host-community turnover, and redox-driven metal speciation, remain hypotheses warranting validation through time-course studies integrating metaviromic, metatranscriptomic, and metabolomic measurements.

In conclusion, this study provides the first comprehensive metaviromic characterization of duck litter treated with compound microbial intelligent membrane fermentation. Our results demonstrate that this technology effectively reduces viral abundance, particularly RNA viruses, and significantly decreases the loads of *ARGs, MRGs, BRGs*, and *VFs*. RNA viruses, especially *Pisuviricota*, emerge as sensitive bioindicators for ecological health assessment in litter environments. This antibiotic-free fermentation system represents a promising strategy for improving duck health and welfare while curbing the dissemination of antimicrobial resistance at its source in poultry production systems. Future mechanistic studies employing time-resolved multi-omics approaches are warranted to validate this framework and to optimize fermentation parameters for maximal mitigation of biological risks.

## Data Availability

The datasets presented in this study can be found in online repositories. The names of the repository/repositories and accession number(s) can be found in the article/supplementary.

## Supplementary material

Supplementary Table 1 Viral gene annotation statistics from the single-sample in-depth metavirome analysis.

Supplementary Table 2 Detailed annotation of antibiotic resistance genes (ARGs), metal resistance genes (MRGs), biocide resistance genes (BRGs), and virulence factors (VFs) identified in the representative duck litter sample.

Supplementary Figure 1 Viral taxonomic composition at family and genus levels **(A)** Pie chart showing family-level viral taxonomy. Caudovirales_norank represents the dominant family (84.6%), followed by unclassified viruses (11.1%) and other families (4.3%). **(B)** Horizontal bar chart displaying the top 15 viral genera ranked by abundance (RPKM × 10³). Caudovirales_norank is the most abundant genus, markedly exceeding other genera.

Supplementary Figure 2 KEGG functional pathway annotation **(A)** Pie chart of KEGG Level 1 functional categories. Genetic Information Processing accounts for the largest proportion (56.8%), followed by Metabolism (27.9%), Human Diseases (4.0%), Environmental Information Processing (2.2%), Cellular Processes (5.3%), and Organismal Systems. **(B)** Bar chart of KEGG Level 2 pathways, with Translation showing the highest abundance. **(C)** Bar chart of the top 25 KEGG Level 3 pathways; Ribosome is the most abundant pathway.

Supplementary Figure 3 Quality assessment of viral operational taxonomic units (vOTUs) **(A)** Pie chart of CheckV quality classification. The majority of contigs are classified as “Not-determined” (n=113, 76.9%), followed by low-quality (n=33, 22.4%) and medium-quality (n=1, 0.7%) contigs. **(B)** Genome completeness distribution of assessed vOTUs; one vOTU reaches >90% completeness (10% threshold indicated by red dashed line). **(C)** Scatter plot of contig length across three quality tiers (Not-determined, Low-quality, Medium-quality), with horizontal lines indicating median length.

Supplementary Figure 4 Viral community composition across taxonomic ranks **(A)** Phylum-level viral composition pie chart; Uroviricota dominates (86.5%), with Hofneiviricota (0.85%) and unclassified viruses (11.1%) as minor fractions. **(B)** Class-level composition bar chart, dominated by Caudoviricetes. **(C)** Order-level composition pie chart, with Caudovirales as the predominant order. **(D)** Top 15 viral species ranked by abundance (RPKM × 10³).

Supplementary Figure 5 Functional gene annotation based on COG and GO databases **(A)** COG functional categories ranked by abundance (RPKM × 10³). **(B)** Top 10 COG categories with functional descriptions. **(C)** Pie chart of GO annotation distribution across three namespaces: biological process (49 genes, 47.6%), cellular component (34 genes, 33.0%), and molecular function (20 genes, 19.4%). **(D)** Bar chart of GO Biological Process terms at Level 2, with cellular process and metabolic process being the most enriched terms.

Supplementary Figure 6 Profiling of antibiotic resistance genes (ARGs), metal resistance genes (MRGs), biocide resistance genes (BRGs), and virulence factors (VFs) **(A)** Pie chart of ARG resistance mechanisms; efflux pump is the predominant mechanism (57.3%), followed by others (25.0%) and mutation (17.7%). **(B)** Bar chart of MRG metal types; iron/copper/manganese resistance is most abundant. **(C)** Bar chart of BRG biocide resistance subtypes, with multi-biocide resistance being dominant. **(D)** VF categories ranked by abundance; nutritional/metabolic factors and immune modulation are the top two categories. **(E)** Combined comparison of gene counts (bars) and total abundance (diamond line) across ARG, MRG, BRG, and VF categories. **(F)** Catalog of key resistance and virulence genes with detailed annotations.

Supplementary Figure 7 A schematic diagram showing the four treatment groups, sample collection workflow (n=5 per group, total N = 20), and the complete analytical pipeline from preprocessing through sequencing, assembly, viral identification, and functional annotation.
